# A new genus of Compositae (Eupatorieae, Piqueriinae) from Peru, named *Centenaria* to honour the 100 ^th^ anniversary of the Natural History Museum of the National University Mayor of San Marcos

**DOI:** 10.3897/phytokeys.113.28242

**Published:** 2018-12-07

**Authors:** Paúl onzáles, Asunción ano, Harold Robinson

**Affiliations:** 1 Laboratorio de Florística, Departamento de Dicotiledóneas, Museo de Historia Natural - Universidad Nacional Mayor de San Marcos, Avenida Arenales 1256, Lima-14, Peru; 2 Instituto de Investigación de Ciencias Biológicas Antonio Raimondi, Facultad de Ciencias Biológicas (UNMSM), Avenida Germán Amezaga 375, Lima 1, Perú; 3 Department of Botany, MRC 166, NMNH, P.O. Box 37012, Smithsonian, Washington, DC. 20013–7012, USA

**Keywords:** Asteraceae, *
Ellenbergia
*, Eupatorieae, *
Ferreyrella
*, Rupac

## Abstract

A little herb from central Peru is recognised as a new species of a new genus. *Centenariarupacquiana* belongs to the tribe Eupatorieae, subtribe Piqueriinae. It has asymmetrical corollas with two inner lobes smaller, a flat and epaleaceous receptacle and the presence of pappus. In Peru, *Centenaria* is related to the genera *Ferreyrella* and *Ellenbergia*, but *Ferreyrella* is different by having no pappus and a paleate receptacle; and on the other hand, *Ellenbergia* is different by having symmetrical corollas.

## Abstract (es)

Una pequeña hierba del centro del Perú es reconocida como una especie nueva en un nuevo género. *Centenariarupacquiana* es miembro de la tribu Eupatorieae subtribu Piqueriinae. Tiene corolas asimétricas con dos lóbulos internos más pequeños, receptáculo plano y sin palea, y presencia de papus. En Perú, *Centenaria* está relacionada a los géneros *Ferreyrella* y *Ellenbergia*, Pero *Ferreyrella* se diferencia por carecer de papus y presentar un receptáculo con paleas; por otro lado, *Ellenbergia* se diferencia por sus corolas simétricas.

## Introduction

The Compositae has more than 30000 accepted species in more than 1900 genera (Nordenstam et al. 2009). Currently, twelve subfamilies are recognised, of which Asteroideae (Cass.) Lindl. comprises over 60% of the species in the family, placed in ca. 1229 genera and 20 tribes and is the largest subfamily of Compositae (Pelser and Watson 2009). One of these tribes is Eupatorieae Cass., the fourth largest tribe in the subfamily and the sixth largest in the family (Funk et al. 2009, Pelser and Watson 2009). The tribe Eupatorieae contains 19 subtribes, 185 genera and 2200 species (as of Hind and Robinson 2007, Rivera et al. 2016); and one of these subtribes is Ageratinae Less. with 26 genera and between 300 and 350 species (Hind and Robinson 2007, Robinson et al. 2009), although these numbers of genera and species will certainly change since, recently, it has been shown that the earlier concept of Ageratinae was polyphyletic (Rivera et al. 2016). For this reason, the subtribe Piqueriinae is treated here as separate (following Robinson et al. 2009).

In Peru, the tribe Eupatorieae is one of the largest with 46 genera and 325 species (Dillon and Sagástegui 2002). The subtribe Ageratinae contains seven genera in Peru (Brako and Zarucchi 1993, Ulloa Ulloa et al. 2004, Hind and Robinson 2007), of which four belong to the narrower concept of Piqueriinae and three (*Ascidiogyne* Cuatrec., *Ellenbergia* Cuatrec. and *Ferreyrella* S.F. Blake) are endemic to this country (Beltrán et al. 2006).

On 15 April 2018 during a field trip, as part of a botanic course of the “Universidad Nacional Mayor de San Marcos”, to an area east to the city of Lima, we found a little plant of Compositae growing along the trail between Pampas and Rupac (Lima, Peru). It was here that serendipity brought us and the new species face to face. After two weeks, we concluded that it was a member of what is here treated as Piqueriinae that had not been previously reported for the Peruvian flora (Brako and Zarucchi 1993, Ulloa Ulloa et al. 2004, 2017, Beltrán et al. 2006, Dillon and Sagástegui 2002, Vásquez et al. 2002, Linares et al. 2010, Gonzáles et al. 2016). For this reason, on 7 May, we decided to make a new trip to look for more populations and to make more collections for the study of this plant.

This previously unknown dwarf member of the tribe Eupatorieae from the higher elevations of the Department of Lima in Peru shows a combination of characteristics unmatched in other previously known genera of the tribe. The entity is described here as the new genus *Centenaria* in honour of the centennial of the establishment of the Natural History Museum of National University Mayor of San Marcos (28 Feb 1918).

As shown in the study of the tribe Eupatorieae of the Asteraceae by King and Robinson (1987), the tribe contains many genera with a dwarf habit and a reduced or absent apical appendage on the anther. These genera are scattered geographically from Mexico southwards to the Andes of South America and eastwards to Brazil. Most genera show ex-imbricate involucres with broad and blunt-tipped phyllaries and corollas with narrow bases and broadly campanulate limbs. Such genera seem mostly to be related to the *Piqueria* group in the subtribe Piqueriinae of the Eupatorieae (Robinson et al. 2009, Rivera et al. 2016). The new genus differs from all of these genera by the presence of a pappus of lanceolate scales on only the inner florets of the capitulum. Zygomorphic corollas of the plant are an unusual characteristic in the tribe, but are also seen in the genus *Microspermum* Lagasca of Mexico, in one of the species of *Iltisia* S.F.Blake of Costa Rica, in one of the species of *Ferreyella* of Peru and in the following members of the monophyletic subtribe Praxelinae (Rivera et al. 2016), the Brazilian and Bolivian turf-forming genus *Piqueriopsis* G.M.Barroso, the Brazilian *Eitenia* R.M.King & H.Rob. and a few members of the mostly Brazilian polyphyletic *Praxelis* Cass. (Rivera et al. 2016). The latter genera can be distinguished by having no pappus or a pappus of many capillary setae. Two genera, *Pigueriopsis* R.M.King of Mexico and *Iltisia* of Costa Rica are distinct in having 4-lobed corollas. Of the above genera, the more northern *Microspermum* and *Iltisia* also seem to have a more remote relationship because of their narrower and more pointed phyllaries.

Of most interest for purposes of comparison are the presumably related dwarf genera that occur in Peru, *Ascidiogyne*, *Ellenbergia*, *Ferreyrella* and *Guevaria* R.M.King and H.Rob. Of these, *Ferreyrella* differs by its paleate receptacle, *Ellenbergia* differs by its pappus of many narrow segments, *Guevaria* differs by its total lack of pappus, its conical receptacle and prostrate to procumbent habit and *Ascidiogyne* differs by its carnose nature and prostrate habit with clusters of short peduncles arising from short leafy lateral branches. These genera are distinguished more completely in the key below.

## Material and methods

All morphological characters were studied under a Leica-EZ4 1×–4.5× stereo microscope and an Olympus SP5-70UZ Digital Camera. Specimens of Piqueriinae housed at herbaria GOET, MO, MOL, P, GH, U, UC, US and USM, (Thiers 2018) have been examined. Digitised specimens were viewed via online herbarium catalogues (Herbarium virtual austral Americano 2018) or via JSTOR (2018).

## Taxonomic Treatment

### Centenaria

Taxon classificationPlantaeLituolidaLituolidae

P.Gonzáles, A.Cano & H.Rob. gen. nov.

urn:lsid:ipni.org:names:77192419-1

#### Description.

Small, erect, annual herbs, to 30 cm tall. Leaves opposite, blade ovate to broadly elliptical, coarsely to finely serrate. Inflorescence a diffuse corymbose cyme. Phyllaries 5, distant, biseriate, subequal to equal, persistent, oblong-elliptical to obovate-elliptical, with shortly apiculate apices; receptacle flat, foveolate, glabrous, epaleaceous. Florets 7–14; corollas asymmetrical with the two inner lobes smaller, white, with distinct short constricted basal tube bearing glandular or eglandular hairs; throat short and broad-campanulate; lobes as long as wide or outer lobes of peripheral florets longer, short-papillose on inner surface and margins; lower part of filament glabrous; anther collars rather short; style base not enlarged, glabrous; arms rather short-clavate, densely short-papillose; papillae larger and less dense below clavate tip. Achenes prismatic, 5-ribbed, ribs setuliferous, narrowed and setuliferous above carpopodium; carpopodium inconspicuously, short-cylindrical; pappus 5 long, lanceolate squamellae, densely scabrid on margins, nearly smooth on outer surface, smooth on inner surface.

### Centenaria rupacquiana

Taxon classificationPlantaeLituolidaLituolidae

P.Gonzáles, A.Cano & H.Rob. sp. nov.

urn:lsid:ipni.org:names:77192421-1

Figure 1

#### Diagnosis.

A little herb characterised by its asymmetrical corollas with two small inner lobes, flat and epaleaceous receptacle and presence of pappus.

#### Type.

Peru. Dept. Lima: prov. Huaral, Dist. Atavillos Bajo, NE of Pampas, road to Rupac, archaeological monument pre Inca, slopes with loamy clay soil, scrubland, −11.313333, −76.61333, 3033–3509 m a.s.l., 15 Apr 2018, (fl,fr), *A. Cano, P. Gonzáles, E. Huamán, S. Riva & S. Rivera 22682* (holotype: USM-3070016!, isotypes: MO!, MOL!, US-3730645!, USM!).

**Figure 1.**
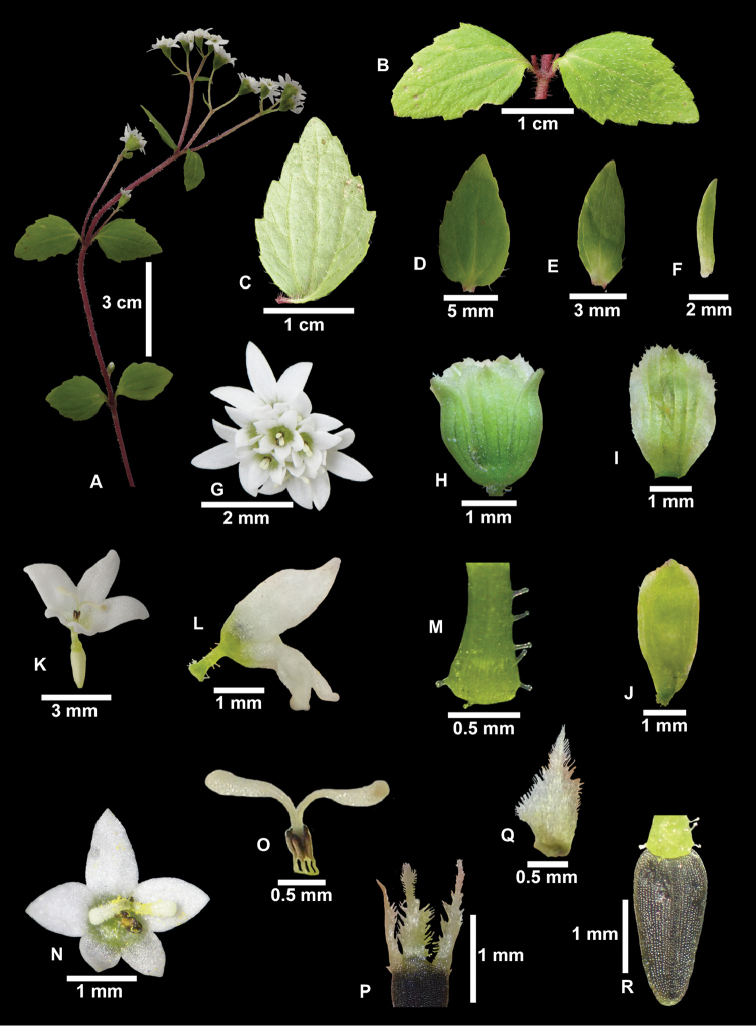
*Centenariarupacquiana*. **A** Plant **B** leaf (adaxial surface) **C** mature leaf (abaxial surface) **D** mature leaf (adaxial surface) **E** upper leaf **F** uppermost leaf bracteiform **G** capitula (upper view) **H** involucre **I** inner phyllaries **J** outer phyllaries **K** flower (frontal view) **L** flower (lateral view) **M** flower tube with glandular trichomes **N** flower (upper view) **O** style and stamens **P, Q** scale of pappus **R** achene without pappus. Photos by P. Gonzáles.

#### Description.

Slender and small herb 9–20(30) cm high, bearing 2–10 heads. Roots thin and delicate, 2–4 cm long. Stem unique, terete, 0.3–1(1.7) mm thick, often purplish tinged, rather densely pilose with several celled acuminate mostly spreading whitish or purplish hair up to 0.3–0.7(1) mm long, internodes 1–3, mostly (1)3–5(6) cm long. Leaves opposite, membranous, petioles short, ca. 1–1.5 mm long, pubescent like the stem, larger leaf blades 10–20 mm long, 7–12 mm wide, obtuse to acutish, at base cuneate, obtusely about 3-toothed on each side, triplinerved from near base and lightly reticulate veiny beneath, pilose on surface above and chiefly along veins beneath with hair like those of stem, thin herbaceous; upper leaves (subtending branches of inflorescence) mostly sessile, smaller, the uppermost bracteiform. Capitula homogama, discoid, mostly in groups of 2 or 10 at tips of stem and branches, 4–5 mm high, 2.5–4 mm thick, on densely pubescent pedicels 2–8(12) mm long, pedicel pilose and pilose-glandulose (globose-stippled glands); involucre 3–3.5(4) mm high, 2–2.3 mm wide, phyllaries 5, biseriate, imbricate, outermost phyllaries 3, membranous, elliptical to obovate-elliptical, obtuse, 3–3.5 mm long, 1–1.7 mm wide, 5-nerved, the phyllaries rather glabrate, apex tinted purple, slightly erose-ciliate, innermost phyllaries 2, elliptical to obovate, obtuse, 3.5–3.8 (4) long, 2–2.2 wide, 5-nerved, the phyllaries rather glabrate, apex erose-ciliate; receptacle flat foveolate; pales none. Flowers hermaphroditic, (7)9–12(14), corollas asymmetrical, the two inner lobes are smaller, white, funnelform, 2 (inner) to 3 (outer) mm long, outermost corollas 3–4 very asymmetrical, the tube 0.9–1.2 mm long, tube base expanded, the throat about 0.3–0.5 mm long, the longer teeth 1–1.5 mm long, triangular-lanceolate, the shorter about 0.2–0.3 mm long, triangular, apex acute, margin stout minute papillose, innermost corollas 7–8(10), the tube 0.7–1 mm long, tube base dilated, the throat about 0.2–0.5 mm long, the longer teeth 0.2–0.3 mm long, triangular-lanceolate, the shorter about 0.1–0.2 mm long, triangular, apex acute, margin stout, minute papillose, anthers oblong, apex obtuse, emarginate, subtruncate, exappendiculate, base obtuse, 0.2–0.3 mm long and 0.1–0.2 mm wide, filaments inconspicuous, ca. 0.1–0.2 mm, stylus crassiusculus, 1–1.3 mm, with 2 stigmatic arms 0.5–0.7 mm long, arms recurvate, clavate, densely papillose in the stigmatic region. Fruit an achene mature black, 1.5–2 mm long, 0.5–0.7 mm wide (above), prismatic, base attenuate, 5-ribbed, ribs setuliferous, narrowed and setuliferous above carpopodium; carpopodium inconspicuous, shortly cylindrical, less than 0.1 mm; pappus (only in the innermost flowers of the capitula, outermost flowers without pappus) of 5 lanceolate squamellae, densely scabrid on margins, nearly smooth on outer surface, smooth on inner surface, scales 1–1.2 mm long, 0.2–0.3 mm wide (lowermost), to 0.7 mm wide (middle part), united in the base, easily separable from the achene.

#### Distribution.

Known only from the type locality in Rupac, northeast from Lima Department.

#### Ecology.

Terrestrial plant growing on open area amongst shrubs, in the western Cordillera shrubland, between 3000–3500 m a.s.l. Co-occurring species include *Paracaliajungioides* (Hook. & Arn.) Cuatrec., *Heliopsisbuphthalmoides* (Jacq.) Dunal, *Dasyphyllumferox* (Wedd.) Cabrera and *Vulpiamegalura* (Nutt.) Rydb. Flowering and fruiting between April and May.

#### Etymology.

The genus is dedicated to the centennial of the institutional foundation of the Natural History Museum of National University Mayor of San Marcos (1918–2018), for their hard work on the research, conservation, preservation and diffusion of the biodiversity of the country. All these actions are steadily increasing our knowledge of the flora and fauna of our native land. The specific epithet refers to Rupac, a small village with archaeological remains from the Atavillos culture, where the only two populations of this species are known from this place.

#### Conservation status.

*Centenariarupacquiana* is only known from the type collection and is therefore assessed as Data Deficient (DD) according to the UICN (2012, 2017) criteria. However, we recommend it should be considered critically endangered (CR), as it is only known from a single locality (Criterion B1a) with a continuing decline of its quality of habitat inferred from the intensive livestock in the area (Criterion B1b). Furthermore, is only known from two populations with an estimated number fewer than 250 mature individuals (Criterion C1a(ii)).

#### Specimens Examined.

**PERU. Lima**: Huaral, Atavillos Bajo, near to village Pampas, road to archaeological monument Rupac, slopes with loamy clay soil, scrubland, −11.323055, −76.78138, 3033–3099 m a.s.l., 7 May 2018, (fl,fr), *A. Cano, P. Gonzáles, E. Huamán & S. Riva 22721* (HUT!, HSP!, MO!, MOL!, SI!, US!, USM-307017!).

#### Discussion.

*Centenaria* belongs to the subtribe Piqueriinae of the Eupatorieae, being considered related to the genera *Microspermum* and *Iltisia* from Mexico and Central America and *Ferreyrella* of Peru, all having asymmetrical corollas with the two inner lobes smaller than the rest; however, *Microspermum*, *Iltisia* and *Ferreyrella* have no pappus or only a few capillary setae (Hind and Robinson 2007). Furthermore, *Centenaria* is evidently related to the genus *Ellenbergia* of Peru, which has a pappus of many segments but has symmetrical corollas.

The species described here is very similar to *Ferreyrella*; both have strikingly asymmetrical corollas, from which it differs primarily in having a flat epaleaceous receptacle (vs. slenderly conic paleaceous receptacle) and the presence of pappus (vs. lack of pappus). Although this distinction is usually important in the *Eupatorieae*, as it is in Compositae generally, it becomes merely a specific or even, in one case, a varietal character in *Ageratum* (Blake 1957). The flat receptacle and the pappus of lanceolate squamellae with densely scabrid margins in *Centenaria* are very suggestive of those of *Ellenbergia*, although the pappus is not so extremely united at the base into a cup-shaped piece, an additional significant feature not found in *Ellenbergia*.

Robinson et al. (2009) commented about a group formed by small, mostly rather ephemeral Eupatorieae, which usually have many-flowered heads. At this time, *Guevaria*, *Ferreyrella*, *Ellenbergia* and *Centenaria* would also fit in this group despite having fewer flowers.

### Key to the dwarf genera of the Eupatorieae in Peru

**Table:** 

1	Rather carnose creeping plants with fascicules of short pedunculate heads on short lateral branches; anthers exappendiculate	*** Ascidiogyne ***
–	Thinly herbaceous-leaved erect or decumbent plants, with heads not in fascicles; anthers with vestigial apical appendages except in *Centenaria*	**2**
2	Receptacles paleate	*** Ferreyrella ***
–	Receptacles without paleae	**3**
3	Achenes without a pappus; receptacles conical	** Guevaria **
–	Achenes with a pappus; receptacles flat or slightly convex	**4**
4	Pappus of many subulate persistent segments; corollas radially symmetrical	*** Ellenbergia ***
–	Pappus of few easily deciduous lanceolate scales restricted to innermost florets of heads; corollas zygomorphic with inner lobes shorter than outer lobes	*** Centenaria ***

## Supplementary Material

XML Treatment for Centenaria

XML Treatment for Centenaria rupacquiana
